# Binocular Depth Judgments on Smoothly Curved Surfaces

**DOI:** 10.1371/journal.pone.0165932

**Published:** 2016-11-08

**Authors:** Rebecca L. Hornsey, Paul B. Hibbard, Peter Scarfe

**Affiliations:** 1 Department of Psychology, University of Essex, Wivenhoe Park, Colchester, CO4 3SQ, United Kingdom; 2 School of Psychology and Clinical Language Sciences, University of Reading, Earley Gate, Whiteknights Road, Reading, RG6 6AL, United Kingdom; University of Alberta, CANADA

## Abstract

Binocular disparity is an important cue to depth, allowing us to make very fine discriminations of the relative depth of objects. In complex scenes, this sensitivity depends on the particular shape and layout of the objects viewed. For example, judgments of the relative depths of points on a smoothly curved surface are less accurate than those for points in empty space. It has been argued that this occurs because depth relationships are represented accurately only within a local spatial area. A consequence of this is that, when judging the relative depths of points separated by depth maxima and minima, information must be integrated across separate local representations. This integration, by adding more stages of processing, might be expected to reduce the accuracy of depth judgements. We tested this idea directly by measuring how accurately human participants could report the relative depths of two dots, presented with different binocular disparities. In the first, *Two Dot* condition the two dots were presented in front of a square grid. In the second, *Three Dot* condition, an additional dot was presented midway between the target dots, at a range of depths, both nearer and further than the target dots. In the final, *Surface* condition, the target dots were placed on a smooth surface defined by binocular disparity cues. In some trials, this contained a depth maximum or minimum between the target dots. In the Three Dot condition, performance was impaired when the central dot was presented with a large disparity, in line with predictions. In the Surface condition, performance was worst when the midpoint of the surface was at a similar distance to the targets, and relatively unaffected when there was a large depth maximum or minimum present. These results are not consistent with the idea that depth order is represented only within a local spatial area.

## Introduction

### Cortical encoding of binocular disparity

Binocular vision provides us with a valuable cue to the three-dimensional shape and location of objects. In order to make use of this information, corresponding points in the left and right eyes must be matched. This allows us to estimate the differences in the location of these points in the two eyes’ images, known as binocular disparities. To achieve correspondence, the brain uses a process that is closely related to the calculation of a local cross-correlation [1,2]. In essence, this involves the comparison of small, local samples from the two images, in order to find the disparity at which the closest match between the left and right eye occurs.

Characterisation of the initial stages of binocular processing in this way has been very fruitful in explaining how we perceive depth from binocular cues. Firstly, it provides a functional account of the receptive field properties of binocular neurons in the primary visual cortex, since these are well suited to the calculation of a local cross-correlation [3–6]. By combining energy responses across orientation, scale and location it is possible to calculate an approximation to a local cross-correlation [3,7,8]. Secondly, the calculations involved in estimating a local cross-correlation impose a number of limitations on the precision with which we can encode variations in depth, including for example the spatial resolution and steepness of depth variations that we are able to perceive [2,9]. A number of adaptations of a standard correlation model, such as a consideration of the bandpass spatial frequency filtering in the visual cortex [10], and the link between correlation window size and disparity [7], have been proposed in order to account for human performance. In order to fully understand the perception of depth, it is also necessary to consider other aspects of disparity processing in the visual cortex.

### Disparity encoding in the extrastriate visual cortex

While the cross-correlation model of disparity encoding has proved very successful in accounting for a number of such properties of binocular depth perception, cortical analysis of binocular information is not confined to this initial processing. Rather, neurons tuned to binocular disparity are found in many visual areas [11,12], and these neurons encode a wide range of depth information. This includes responses to disparity-defined edges in V2 [13,14]; to relative disparities between surfaces in V3/V3A [15] and V4 [16]; to the three-dimensional orientation of surfaces in V4 [17] and V5 [18]; and to surface curvature in the inferotemporal cortex [19]. This reflects the fact that considerable further analysis of cross-correlation responses is needed in order to estimate disparity, and to use this to represent the location, orientation and shape of objects in three dimensions.

The perception of depth from binocular information will therefore be determined by how disparity is processed in all of these areas. Indeed, it is important to note that the perception of depth is more closely related to activity in extrastriate areas than V1, where the initial encoding of binocular information occurs [20,21]. The wide variety of disparity structures encoded across the visual cortex suggests that we might form many different kinds of representations of depth, rather than a single depth map of the three-dimensional location of each point [22]. For example, it has been suggested that we make direct use of information not only about the depths of individual points, but also the 3D orientation and curvature of surfaces [23–27].

### Contextual effects on depth sensitivity

One way to understand these representations is to consider how the perceived three-dimensional location of individual points is determined by their local spatial context. A number of contextual effects have been demonstrated, in which our sensitivity to depth differences between individual points depends on the presence and characteristics of other nearby features. These contextual effects reflect the action of a number of different mechanisms of depth processing.

#### Gestalt grouping and shape recognition

Binocular disparity plays an important role in segmenting the visual image, so that features belonging to distinct surfaces are grouped together. Many other image characteristics, such as the proximity and similarity of individual features, or the continuity and closure of contours, play a similar role [28]. In natural scenes, when multiple grouping cues are available, these will typically be in agreement. For example, if one surface is closer than another, and the two surfaces differ in colour, then grouping by binocular disparity and colour will provide consistent results. This allows cues to be combined cooperatively to improve the accuracy of grouping [29,30]. In psychophysical studies, conflicts with other cues have been shown to decrease our sensitivity to disparity-defined depth [31–33]. These results may be interpreted as the consequence of combining conflicting evidence to the best segmentation of the image into distinct surfaces, rather than any direct effect on the way in which binocular disparity itself it processed. However, Gestalt grouping via closure also reduces the amount of depth that is perceived [34,35]

#### Disparity discontinuities and the perception of stereoscopic depth

Our sensitivity to the depth difference between two vertical lines is greatly diminished if they are joined together to form a rectangle, even though this manipulation does not affect the horizontal disparity information that is available [36–38]. While these results might to some extent reflect conflicts between binocular and perspective cues, similar results have been found with even simpler stimuli [39]. In this latter study, sensitivity to a depth difference between two dots deteriorated if one or more additional, but irrelevant, dots were positioned between them. It was concluded that precise depth judgments can only be made between adjacent points, and that the presence of additional points lying between the two target features disrupts this process.

Other studies have shown that we are sensitive not to the disparities in individual features, but to the relative disparities between them. For example, stereoscopic thresholds are substantially lower when a reference line is presented in a stimulus as well as the target [36–38]. Spatial context also affects sensitivity to more complex variations in depth. When individual points lie on a surface that is slanted in depth, a spatial gradient of binocular disparity is present. This gradient reflects the relative disparities between points that are not at the same distance. When such a surface is presented in isolation, we are relatively insensitive to its slant [40,41]. In this case, our perception of slant is greatly enhanced when another surface is presented in a way that produces a gradient of relative disparity. This is evident in an increase in apparent slant [40, 42–47] and a decrease in the time taken for it to be seen [40–42, 48]. This increase in apparent depth results from gradients of relative disparity provided by both the outline of the surface, and its texture [49].

#### The reference frame for stereoscopic depth

Spatial variations in depth are defined relative to a particular coordinate system. For example, the standard cross-correlation model, by specifying the disparity of points as a function of their horizontal and vertical position in the image, defines depth within a retinotopic coordinate system. However, measurements of our sensitivity to stereoscopic depth suggest that it is in fact represented in a coordinate system defined by a reference plane defined by the local surface structure [50–54].

This surface-based representation requires the coordinate system to be specified in terms of the information available in the local spatial neighbourhood. Psychophysical studies that have clearly demonstrated the importance of a locally defined coordinate system have used a single, planar surface as a reference [38,50–52]. As such, they do not address how this reference surface might be defined in natural scenes, in which the depth variations will be more complex.

One approach to defining the apparent depth of a point relative to others in the local neighbourhood is to calculate its *salience*, defined as the weighted sum of the difference in the disparity of the target and other points in the scene [38]. The weights used in calculating the saliency measure are inversely proportional to the distance between points, and also include a masking factor, to favour neighbouring features that are not separated by some other intervening feature.

A local reference frame might also be created through a hierarchical process, in which depth variations at a given scale are specified relative to a coordinate system defined by information at a coarser scale [55]. Another method is to parse objects into local subregions based on the topographical structure of surfaces, and use the subregions identified in this way to define the local reference frame [56]. This approach segments objects into ‘hill districts’, surrounding local depth maxima, separated by ‘ruts’ defined by the local depth minima.

**Fig 1:**
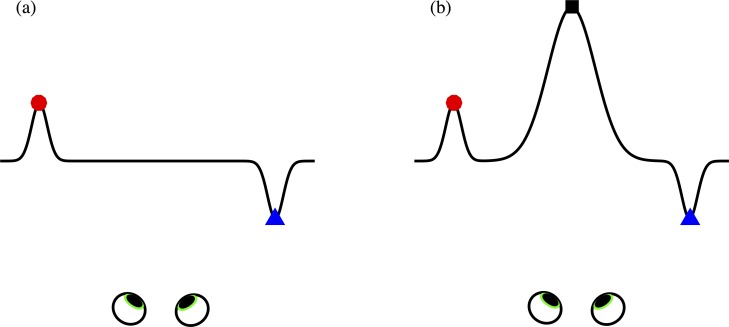
Examples of monotonic and non-monotonic depth relationships. **(a)** An otherwise frontoparallel surface with a dip on the left (marked by the red circle) and a bump on the right (marked by the blue triangle). **(b)** The same surface, with the same dip and bump, which are now separated by a large central dip (indicated by the black square). The depth relationship between the red and blue points in monotonic in **(a)**, but not in **(b)**.

This parsing of the scene into distinct subregions could potentially account for contextual effects on the ability of observers to make accurate judgments of ordinal depth. In an ordinal depth discrimination task, the observer is required simply to say which of two targets is the closer. The accuracy of ordinal depth judgments on simple surfaces defined by shading or texture cues is greater when depth varies monotonically between two points, than when the points are separated by a local maximum or minimum in depth [57]. Similarly, it has been reported that, when comparing nearby points, observers can make reliable ordinal depth judgments in photographs, and that these judgments are consistent with a well-defined surface relief. However, when the points were widely separated in the picture plane, performance was unreliable [58]. It was suggested that this arises as we are able to make reliable judgments for points lying on a single slope in depth, but not for points that are separated by a depth maximum or minimum (Fig 1). This local slope can be defined with reference to the local depth maxima and minima. As the distance between the points in a natural scene increases, they are more likely to be separated by a maximum or a minimum, thus decreasing our sensitivity to their relative depth order. It is also possible that, rather than observers being completely unable to make reliable depth judgements when points are separated by a maximum or minimum, their sensitivity might be impaired in this case.

The manner in which the scene is parsed into local subregions could also account for a similar deterioration in ordinal depth judgments that has been found for points lying on the surface of a smooth object, defined by shading, texture and stereo cues [59]. Thresholds were measured for determining which of two points, presented in empty space, was the closer to the observer. When the task was changed so that the two points were presented on a smoothly curved surface, observers were significantly worse at determining which of the two was closer. It was concluded that our knowledge of the ordinal depth relationships between points is limited to local spatial neighbourhoods.

Fig 1A shows a surface that is frontoparallel to the observer, apart from a dip on its left-hand side (marked by the red circle) and a corresponding bump on its right-hand side (marked by the blue triangle). The distance from the observer decreases monotonically as we move from the red circle to the blue triangle. A similar surface is presented in Fig 1B. The red and blue points are in the same locations, but there is now a large dip in the centre of the surface, marked by a black square, such that the points are now separated by a depth maximum. While the red circle is still further from the observer then the blue triangle, both are now close relative to the black square.

### Goal of the current study

The purpose of the current study is to establish how the initial encoding of disparity constrains the way in which depth is represented in more complex scenes, such as those containing multiple objects and surfaces. It is clear from previous studies that, as we extend our consideration from very simple stimuli to those with a more complex surface structure, it is not straightforward to predict how accurately people will be able to perceive variations in depth. If we are able to directly compare the depths of the two target points, then the presence of an intervening depth maximum or minimum (Fig 1) should not affect our ability to say which is closer. However, if our representation of ordinal depth relationships is confined to local spatial neighbourhoods [57–59], and if these local neighbourhoods are determined by the locations of local maxima and minima in depth [56], then the presence of an intervening depth maximum or minimum would be expected to lead to a deterioration in performance. Our goal was to determine whether a parsing of surfaces into regions defined by maxima and minima in depth, and the use of these regions to define a local reference surface, can account for observers’ sensitivity to ordinal depth differences.

To assess this, we measured depth discrimination thresholds for two dots presented in empty space, in front of a background grid, and again when these dots were separated by a third irrelevant dot, or were placed on a smooth surface. The location of this central dot, and the shape of the surface, were systematically varied so as to place a depth maximum or minimum between the targets dots. While this comparison has been made for surfaces defined by shading and texture [57] it has not been tested directly for surfaces defined by binocular disparity. Rather, those studies that have varied the local 3D context to assess its effect on stereoscopic depth sensitivity have tended to focus on slanted planar surfaces [38, 50–54]. We predicted that, when the dots lay on a single slope, and were not separated by a depth minimum or maximum, depth sensitivity would be greatest, and that sensitivity would decrease as the disparity of the central point increased. In contrast we found that, while the presence of a single dot, or a smooth surface, did impair performance, in the presence of a smooth surface sensitivity to ordinal depth differences was worst when the depth variation on the surface was at a minimum.

## Experiment One Materials and Methods

### Ethics statement

All procedures were approved by the University of Essex Ethics Committee. Participants provided written informed consent to participate in the study.

### Participants

16 participants (9 male, 7 female) completed the experiment, including authors RH and PH. The other 14 participants were naïve as to the purpose of the study.

### Apparatus

Stimuli were presented on a VIEWPIXX 3D monitor, viewed from a distance of 130cm. The monitor screen was 52cm wide and 29cm tall. The screen resolution was 1920x1080 pixels, with a refresh rate of 120Hz. Each pixel subtended 0.72 arc min. Stimuli were presented at 8-bit resolution. Stereoscopic presentation was achieved using a 3DPixx IR emitter and NVIDIA 3D Vision LCD shutter glasses. The crosstalk between the left and right eyes’ views, as measured with a Minolta LS110 photometer, was 0.12%. Participants’ responses were recorded using a RESPONSEPixx response box. Stimuli were generated and presented using MATLAB and the Psychophysics Toolbox extensions [60–62].

### Stimuli

#### Two Dot Condition

**Fig 2:**
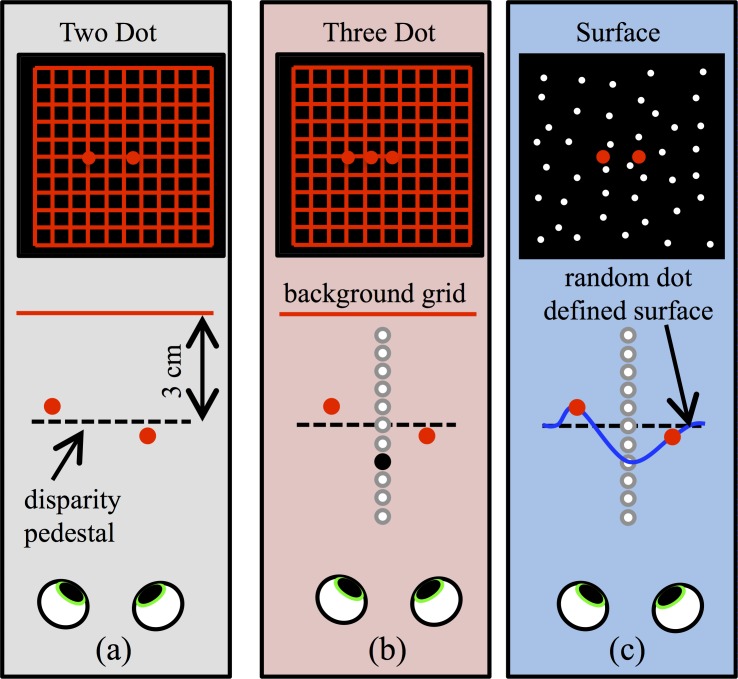
Diagrams to illustrate the stimuli. **(a)** In the Two Dot condition, the two target dots were presented in front of a square background grid. The pedestal depth was 3cm **(b)** In the Three Dot condition, an additional dot (shown here in black in the bird’s eye view) was presented midway between the two target dots. This was presented at 11 possible positions in depth relative to the pedestal. These are indicated here by the unfilled circles. **(c)** In the Surface condition, the target dots were presented against a smooth surface, defined by a random dot stereogram. The surface was constrained to pass through the locations of the target dots, and the same central positions at which the central dot was placed in the Three Dot condition.

This experiment was based on experiment one in [59]. A red square grid (9.5x9.5 degrees) was presented as a background surface. This was centred at eye height and directly in front of the participant, with zero disparity relative to the screen. The grid consisted of 11 horizontal and vertical red lines, 1 pixel wide and 9.5 degrees long, with a separation between the lines of 57 arc min. The target stimuli consisted of two circular red dots, each with a diameter of 14.3 arc min, presented with a crossed pedestal disparity of 10.7 arc min, so that they appeared 3cm in front of the grid (Fig 2A). The dots were separated horizontally. In two separate blocks of trials, this separation was 36 or 72 arc min. Unlike in the previous study [59], the grid and dots were presented in the same colour, to eliminate other cues to segmentation.

The dots were presented at eye height, and their horizontal position was randomised on each trial, within a 72 arc min region around the centre of the grid. On each trial, one of the two dots (chosen at random) was given an additional crossed disparity relative to the pedestal, and the other an uncrossed disparity of the same size. Within each block of trials, 11 values of relative disparity were presented 20 times. The participant’s task on each trial was to determine whether the left or right dot was closer in depth. At least one block of trials was completed by each observer, and the experiment was used to calculate a depth discrimination threshold for each participant at each separation.

#### Three Dot Condition

The stimulus was identical to that used in the Two Dot condition, except for the addition of a third red dot centred between the other two (Fig 2B). The separation of the outer two dots was 72 arc min, and the separation between the centre dot and each of the target dots was 36 arc min. These distances therefore matched the two separations used in the first experiment. Across separate blocks of trials, the disparity of the central dot, relative to the pedestal, was varied. For each participant, this additional disparity was a multiple of their discrimination threshold from the Two Dot condition, for a horizontal separation of 72 arc min. This allowed us to assess the effect of the presence of the central dot on the participant’s depth discrimination threshold. The additional disparities used for the central dot were 0, or ±0.5, 1, 2, 4 or 8 times the discrimination threshold, giving a total of 11 levels (Fig 2B). This created a number of arrangements of the three dots in depth: (i) both target and reference were further away than the central dot, (ii) the central dot lay between the target and reference dots in depth and (iii) both the target and reference dots were closer than the central dot. Within a single experimental block, the central dot had a constant disparity. We predicted that, when the depth did not increase or decrease monotonically across the three dots (arrangements (i) and (iii)), depth sensitivity would deteriorate. We thus expected depth discrimination thresholds to increase as the disparity of the central dot increased. As in the Two Dot condition, 11 values of relative disparity between the left and right dot were each presented 20 times, in random order, within a block of trials. At least one block was completed by each participant in each condition, and additional blocks were added if this did not create a clear psychometric functions. Data were combined across blocks to measure a threshold for discriminating the relative depth of the reference and test dots, in the presence of the central dot.

#### Surface Condition

**Fig 3:**
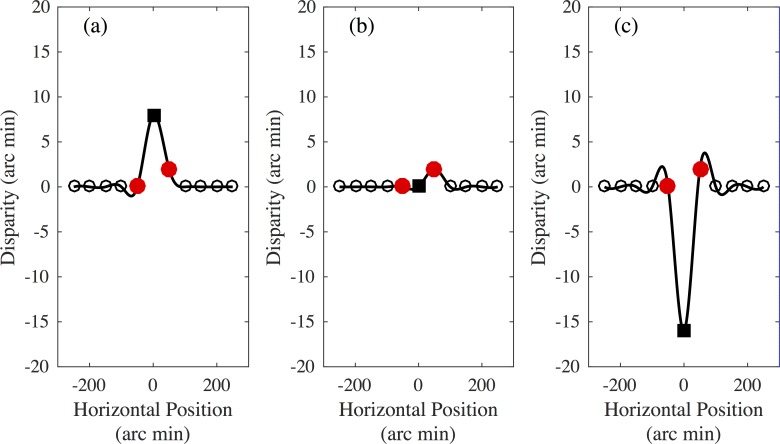
The depth profile of the random dot surface. This was determined by interpolating a spline through a set of references points. Two of the points, indicated by red circles, were the 3D locations of the targets. A third point, indicated by a black square, was in the centre of the surface, at a disparity that was a multiple of the participant’s depth discrimination threshold, as described in detail in the text. The remaining points were evenly spaced horizontally, and all at the same pedestal disparity. **(a)** A surface with a small dip in the centre. **(b)** A surface in which the central point is at the pedestal disparity. **(c)** A surface with a large bump in the centre.

The presentation of the two target dots was identical to that in the Two Dot and Three Dot conditions. However, in this condition they were presented on a surface defined by a random dot stereogram (RDS), rather than the background grid (Fig 2C). The dots (20000 white circlular dots with a diameter of 1.4 arc min) were randomly positioned within an 11.9x11.9 degree square region, giving a density of 142 dots/degree^2^, positioned with sub-pixel accuracy using hardware antialiasing. The disparity of the surface varied in the horizontal direction. To determine the shape of the random dot surface, a smooth spline was interpolated between 11 pre-specified positions in depth (Fig 3). These points consisted of 11 horizontal locations, evenly spaced with a separation of 71.6 arc min, covering an 11.9 degrees range around the centre of the screen. The central three of these positions coincided with the locations of the target dots, and the centre of the random dot surface.

On each trial, the locations on the random dot surface that corresponded to the positions of the two target dots were given depth values that equalled those of the targets. The depth of the centre of the random dot surface was set at a multiple of the participant’s depth discrimination threshold, in the same way as for the Three Dot condition. The positions in depth of the other 8 locations on the random dot surface were all fixed at the crossed pedestal disparity of 10.7 arc min. The depth of the surface was constant at all vertical positions. A smooth depth profile was then interpolated between the points with a cubic spline interpolation, using the MATLAB *spline* function. This produced a smooth surface that passed through the 3D locations of the target dots, and a pre-specified location in the centre of the surface. The resulting surface was vertically oriented and corrugated in depth in the horizontal direction. The target dots were positioned so as to occlude the surface dots, and appeared as two red dots upon the corrugated surface.

Within a single experimental block, the central point on the surface had a constant disparity, and each block was used to measure a threshold for discriminating the relative depth of the two test dots. As in the Three Dot condition, the additional disparities used for the central position of the surface were 0, or ±0.5, 1, 2, 4 or 8 times threshold (Fig 2C). This produced a surface with a central bump (positive disparities) or dip (negative disparities). We predicted that the sensitivity to the depth difference between the target dots would decrease with the size of the bump or dip. Eleven relative disparities between the target dots were each presented 20 times, in a randomised order, within each block of trials. For each central disparity, at least one block was used to measure a threshold for discriminating the relative depth between the two target dots, when presented on a smoothly curved surface. The maximum disparity gradient on the surfaces, across all stimuli presented, was 0.85, below the proposed disparity gradient limit of 1 [63]. The Nyquist limit for the dot density used was 5.9 cycles/degree. At this frequency, the separation between the peak and trough of the depth corrugation would be 6.1 arc min. The 36 arc min separation used here is thus nearly 6 times larger than this sampling limit.

### Procedure

The experiment used a repeated measures design. All participants completed the Two Dot condition first, to determine their threshold, followed by the Three Dot condition and finally the Surface condition. Within each experiment, the order of blocks of stimuli was randomly determined for every participant. The experiment took place over a number of sessions. The participants’ task was to determine which of the two dots appeared closer in depth and indicate this by pressing the corresponding button on the response box. The stimulus was presented until the participant made a response, with an unlimited viewing time. The stimulus was removed once a response was made, with a delay of one second before the next stimulus appeared.

A cumulative Gaussian curve was fit to participants’ responses, using the Palamedes toolbox [64], with threshold (α) and slope (β) as free parameters. The depth discrimination threshold was defined as half the difference between the 25% and 75% points on this curve. The depth discrimination thresholds from the Two Dot condition were used to determine the disparity of the central dot in the Three Dot condition, or the location in depth of the central point of the surface in the Surface condition, as detailed above.

## Experiment One Results

### Two Dot Condition

**Fig 4:**
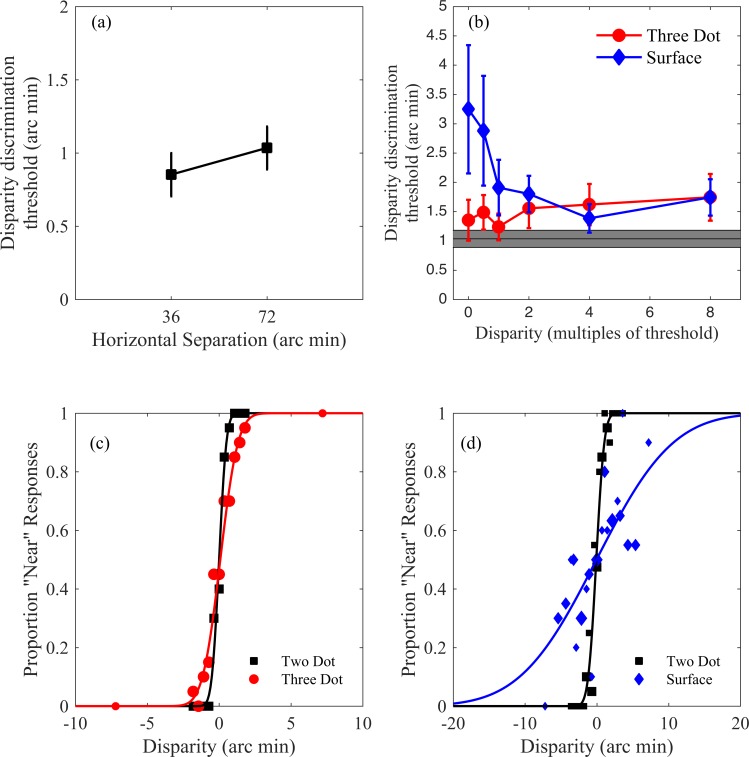
**Experiment one results (a)** Mean disparity discrimination thresholds for the Two Dot condition, for the two separations used. Error bars show ±1 SEM. **(b)** Thresholds for the Three Dot and Surface conditions, as a function of the disparity of the central dot, or the centre of the random dot surface. The grey rectangle plots the mean and standard error of the threshold for the two-dot condition, with a separation of 72 arc min, for comparison. **(c)** Example psychometric functions for the two-dot and three-dot conditions **(d)** Example psychometric functions for the two-dot and surface conditions. Marker areas are proportional to the number of trials.

The mean 75% disparity discrimination thresholds, across all participants, are plotted for the two horizontal dot separations in Fig 4A. Curve fits accounted for 84% of the variance on average. Thresholds were larger for the larger separation (t(15) = 4.291; p<0.001), consistent with previous findings [59]. These thresholds were used to determine the position of the central dot, or the central point of the interpolated surface, in the Three Dot and Surface conditions.

### Three Dot and Surface conditions

For each disparity of the central position, a disparity discrimination threshold was calculated, for both the Three Dot and the Surface conditions. Curve fits accounted for an average of 91% of the variance for both conditions. Example psychometric functions for each condition are plotted in Fig 4C and 4D. Thresholds, averaged over crossed and uncrossed disparities and across all observers, are plotted in Fig 4B. These thresholds were analysed using two mixed effects linear models, for each of the Three Dot and Surface conditions. Each model consisted of disparity sign (whether the centre of the stimulus had a crossed or uncrossed disparity) as a fixed factor, the log of disparity magnitude as a fixed covariate, and random intercepts.

In the Three Dot condition, disparity thresholds increased significantly with the disparity of the central dot (slope = 0.129; t(1,157) = 2.03; p = 0.0436) but was not affected by the sign of this disparity (t(1,157) = 0.029; p = 0.977). This result is consistent with our predictions, in that the threshold increased as the disparity of the central target increased. For the Surface condition, disparity thresholds decreased significantly with increasing disparity in the centre of the surface (slope = -1.120; t(1,157) = -2.00; p = 0.047), but were not affected by the sign of the disparity (t(1,157) = -1.10; p = 0.275). The results are in the opposite direction to the predicted effect. As is clear from Fig 4B, poorest discrimination occurred when the centre of the surface was at the pedestal disparity, and the variation in depth on the surface was at a minimum.

## Experiment One Discussion

We measured sensitivity to ordinal depth differences defined by binocular disparity, for isolated points and for points on smoothly curved surfaces. Consistent with previous reports [39] we found that the presence of even a single dot could disrupt performance on this task, but that placing the points on a smooth surface was most disruptive, particularly when this surface was close to being planar. For points on a smooth surface, ordinal depth judgments were more accurate on surfaces with large variations in depth than on those that were close to frontoparallel.

In the Two Dot and Three Dot conditions, a frontoparallel grid was presented 3 cm behind the depth pedestal of the target dots (with a relative disparity of 10.7 arc min between the mean of the dot disparity and the grid). This grid was not present in the surface condition. This was presented so as to match the stimulus configuration used in a previous study [59]. It is possible that the grid itself might have affected the participants’ sensitivity to depth differences between the target dots. It could, for example, improve performance by providing a frontoparallel reference surface against which judgments could be made. The effect of a frontoparallel reference plane on sensitivity to a relative disparity difference has been assessed in a number of other studies. When the background is presented at the same disparity as one of the targets, it has been found to improve sensitivity [65]. When presented with an uncrossed disparity of 4 arc min, with targets presented on the fixation plane, a background surface was found to increase sensitivity for one observer, and to have no effect for one other [50]. When the targets were presented on the fixation plane, and the reference had a crossed or uncrossed disparity of 10 arc min, it reduced sensitivity to the relative disparity in the targets [65]. From these results, we may conclude that a frontoparallel reference plane increases sensitivity to depth differences when it is close to one of the stimuli in depth, but impairs performance when presented at a different depth. It is important however to note that in all these studies the stimuli were briefly presented, for 150ms, with fixation carefully controlled. This contrasts with the unlimited presentation time and free viewing used in the current study. It is therefore difficult to predict, from previous studies, what effects of the background grid might have had in our experiments. We therefore directly assessed the effect of the background grid on performance in the two-dot three-dot condition in an additional experiment.

## Experiment Two Materials and Methods

### Participants

15 participants completed the experiment, including authors PH and PS. The other 12 participants were naïve as to the purpose of the study.

### Apparatus

The apparatus were the same as used in experiment one.

### Stimuli

The target stimuli consisted of two circular red dots, each with a diameter of 14.3 arc min, presented with a pedestal disparity of 10.7 arc min. The dots were separated horizontally by 36 or 72 arc min, and were either presented in isolation, or in front of background grid, as in experiment one. Trials from these four conditions were presented in separate experimental blocks.

## Experiment Two Results and Discussion

**Fig 5:**
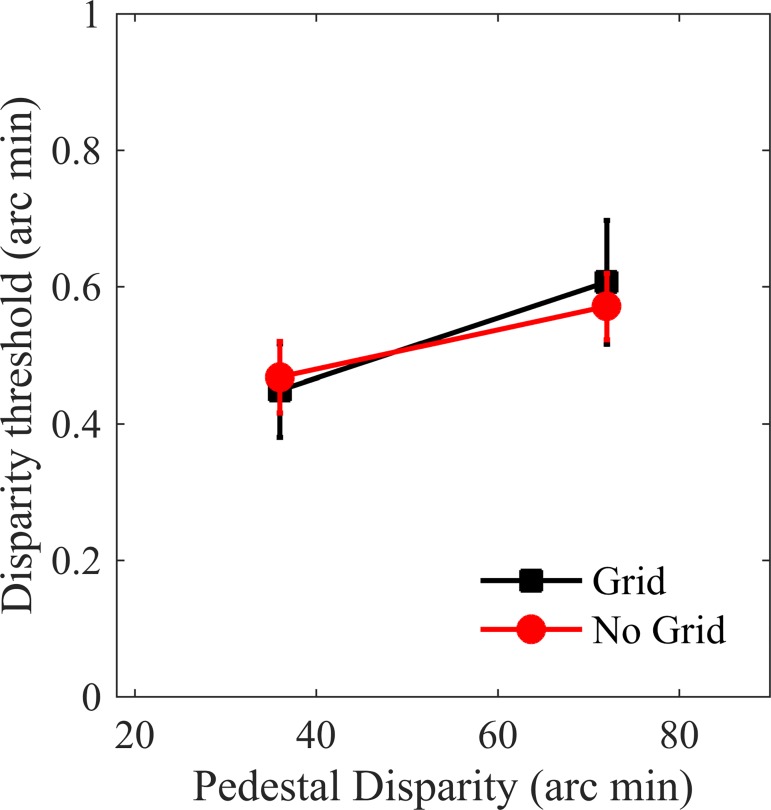
Experiment two results. Mean disparity discrimination thresholds for the Two Dot condition, for the two separations used, both with and without a brackgroun grid. Error bars show ±1 SEM.

Mean 75% correct disparity discrimination thresholds are plotted in Fig 5. There was a significant effect of dot separation (F(1,14) = 17.04; p = 0.001), but no significant effect of the presence of the grid (F(1,14) = 0.011; p = 0.918) and no significant interaction (F(1,14) = 0.576; p = 0.460). These results confirm that the increase in discrimination threshold found in the first experiment did not depend on the presence of the grid, and that the grid itself had no effect on discrimination thresholds.

## General Discussion

Our results demonstrate a clear difference between the way that depth is represented on the basis of binocular and pictorial depth cues. When surface structure is defined purely by pictorial cues, performance on an ordinal depth task is greatly diminished when the points are separated by an intervening maximum or minimum in depth [57,58]. In contrast, we found that performance was best in this condition, and worst when the surface was close to fronto-parallel, with minimum variation in depth. This work extends previous findings that our sensitivity to depth from disparity is diminished by the presence of a local reference defined by disparity, texture and shading, to explicitly test the role of depth maxima and minima in this contextual effect.

Our results are not consistent with any limitations imposed by the cross-correlation stage of processing, which is most accurate when surfaces are close to frontoparallel. They are also not consistent with the idea that relative depth judgments on complex surfaces are imprecise as a result of ordinal depth relationships being represented only over a local spatial neighbourhood [50–55]. If ordinal judgments of depth from binocular disparity were constrained in this way, then performance would also deteriorate in the surface condition, rather than improve, when a depth maximum or minimum was present. For example, if a large bump were present in the centre of the surface, then both target points would be classified as far relative to this point, and also would not lie on the same slope on the surface; it would be predicted that it would not be possible to make an accurate depth comparison across this local maximum.

It has also been suggested that, if depth representations were limited in this way, then depth judgments on curved surfaces might be dependent on information about surface slant [58]. Observers might then rely on the integration of slant across the surface that connects the two points to compare their depth. This process of integrating slant information across a surface would be expected to accumulate errors, and so lead to less accurate performance for more widely separated points [59]. The accuracy of the final relative depth estimates would also depend on the accuracy with which slant is represented. Thresholds for discriminating the difference in surface slant at two locations on a smooth surface have been found to be around 10 degrees, and not to vary with the actual slant of the points compared [66]. From this, we can predict that the errors in relative depth judgments resulting from the integration of slant information should not be affected by the variations in slant on the surface.

Our results can also be considered with respect to the idea that our representation of depth from disparity is defined relative to a local, planar reference [50–55]. This would then mean that the depths of the two target points in our experiment would be defined relative to different coordinate systems. To make a comparison between them would then require the integration of slant information. While a fully specified model of this process would be required to make specific predictions, the similarity of this process to that discussed above [59] means that it is again unlikely to explain why performance was most impaired when the surface was close to frontoparallel.

We also assessed whether our results could be explained by the idea of depth salience [38]. The salience of a feature f_i_, with disparity d_i_ is calculated based on the disparities of neighbouring features:

$$L_{i}=\sum_{j=1}^{n}w_{j}\left(d_{i}-d_{j}\right)$$ (1)

The weights *w*_*j*_ are determined by the distance between the two points, and a potential ‘masking factor’ to reduce the influence when points are separated by other, intervening features. For the three-dot condition, we can calculate the salience of the left and right target dots by considering the disparities and positions of all three dots presented:

$$L_{L}=w_{M}(d_{L}-d_{M})+w_{T}(d_{L}-d_{R})$$ (2)

$$L_{R}=w_{M}(d_{R}-d_{M})+w_{T}(d_{R}-d_{L})$$ (3)

where *L*_*L*,*R*_ are the salience of the two target dots, *d*_*L*,*M*,*R*_ are the disparities of the left, middle and right dots, and *w*_*M*,*T*_ are the weights associated with the difference between the target and middle dots, or the two target dots. The difference in salience between the dots *(w*_*M*_(*d*_*L*_ − *d*_*R*_)*)* does not depend on the disparity of the central dot.

**Fig 6:**
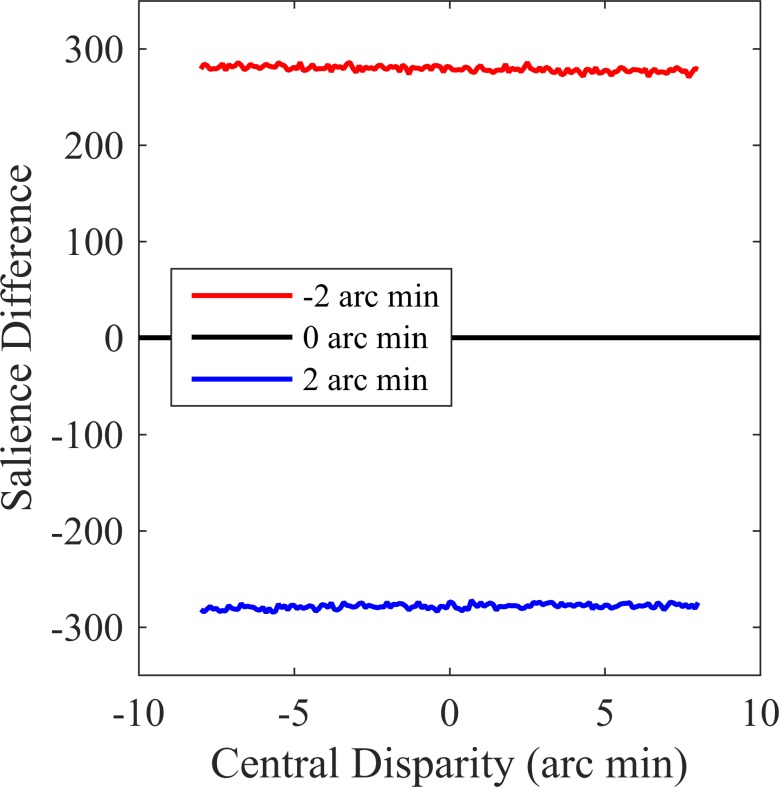
Salience difference calculations for our surface stimuli. The lines show the results for three differences in target disparity, plotted against the disparity of the central bump or dip. Salience was calculated using Eqs 1–3, based on the positions and disparities of all the dots in the stimuli.

For the surface condition, we can also calculate the salience difference by considering all of the dots presented on the surface. This goes beyond the scope of the configurations originally considered [38], whose aim was to understand the perception of depth in simple figures. Nevertheless, Eq 1 can be used to calculate salience for these stimuli. We assumed that weights were inversely proportional to the distance between the points, and did not include a masking factor. Salience difference values are shown in Fig 6. It is clear that the salience difference is determined by the difference in disparity of the two target dots, and is not affected by the manipulations of surface structure used here.

Alternatively, for densely textured surfaces, it may be more appropriate to consider the surface depth variations, rather than the individual texture elements, as the relevant features with which to calculate salience. In this case, the most salient surface feature is the central depth maximum/minimum. The predictions are then the same as those for the three-dot condition, namely that the disparity of the central point will not affect the salience of the difference in disparity of the target dots.

In conclusion, we found that our sensitivity to ordinal depth differences is affected by the local 3D spatial context, consistent with previous results [39]. However, contrary to our predictions, based on previous research, performance was worst for points presented on a surface that was close to frontoparallel. These results cannot readily be explained as resulting from constraints imposed by the encoding of disparity through a process of local cross-correlation, from a representation of depth that is locally defined, or one that depends on access to higher-order information about surface slant. Our results do however demonstrate a clear contextual effect on the perception of depth. Effects of this kind are important in developing an understanding of the way that depth is represented, and how this relates to the processing of binocular information in the visual cortex. They also highlight the difficulty of making predictions based on the conclusions of studies using similar, but simpler stimuli [50–55] or those in which depth information is provided by different cues [57]. The variations in depth in our stimuli are very minimal in comparison with that found in natural results. The results of experiments using simple or reduced cue stimuli must therefore be treated with caution when considering our ultimate goal of understanding perception in the natural environment [67–69].
